# Lifetime total and beverage specific - alcohol intake and prostate cancer risk: a case-control study

**DOI:** 10.1186/1475-2891-3-23

**Published:** 2004-12-09

**Authors:** Maddalena Barba, Susan E McCann, Holger J Schünemann, Saverio Stranges, Barbara Fuhrman, Sabino De Placido, Giuseppe Carruba, Jo L Freudenheim, Maurizio Trevisan, Marcia Russell, Tom Nochajski, Paola Muti

**Affiliations:** 1 Department of Social and Preventive Medicine, School of Public Health and Health Professions, University at Buffalo, Buffalo, NY 14214, USA; 2 Departments of Endocrinology and Oncology, Federico II Medical School, University of Naples, Naples, Italy; 3 Department of Epidemiology, Division of Cancer Prevention and Population Sciences, Roswell Park Cancer Institute, Buffalo, NY 14263, USA; 4 Department of Medicine, University at Buffalo, State University of New York, Buffalo, NY, USA; 5 Department of Preventive Medical Sciences, Federico II Medical School, University of Naples, Naples, Italy; 6 Gynecologic Oncology Group, Roswell Park Cancer Institute, NY, USA; 7 Institutes of Oncology, School of Medicine, University of Palermo, Palermo, Italy; 8 Prevention Research Center, Pacific Institute for Research and Evaluation, Berkeley, CA, USA; 9 School of Social Work, State University of New York at Buffalo, Buffalo, NY, USA

## Abstract

**Background:**

We investigated lifetime alcohol consumption and prostate cancer risk in a case-control study conducted in Buffalo, NY (1998–2001).

**Methods:**

The study included 88 men, aged 45 to 85 years with incident, histologically-confirmed prostate cancer and 272 controls. We conducted extensive in-person interviews regarding lifetime alcohol consumption and other epidemiologic data.

**Results:**

Prostate cancer risk was not associated with lifetime intake of total and beverage specific ethanol. In addition we found no association with number of drinks per day (average drinks per day over the lifetime) or drinks per drinking day (average drinks per day on drinking days only over the lifetime). However, we observed an inverse association with the total number of drinking years. Men in the lowest tertile of total drinking years had a two-fold prostate cancer risk than men in the highest tertile (OR 2.16, 95% CI 0.98–4.78, p for trend <0.05).

**Conclusion:**

Our results suggest that alcohol intake distribution across lifetime may play a more important role in prostate cancer etiology than total lifetime consumption.

## Background

Prostate cancer is the most frequently diagnosed malignancy and the second leading cause of cancer death among men in the Western countries [1]. Notwithstanding the importance of this malignancy, little is understood about its cause. To date the only well established risk factors are age, family history of disease, race and country of residence [2], while the body of the evidence about the role of alcohol intake is still controversial. Since alcohol consumption is a common lifestyle factor and potentially modifiable, the finding of an association with prostate cancer could have an important impact on public health.

Among the population-based case-control studies, those carried out by Heyes et al. [3] and Sharpe et al. [4] found an increased risk of prostate cancer associated with alcohol consumption. Risk increased with increasing frequency of alcohol consumption [3] and among those who drank regularly over a longer period [4]. Sesso et al., in their prospective cohort study, confirmed the finding of a higher risk associated with alcohol consumption [5]. However, numerous studies published since 1998 have not found an association between alcohol intake and prostate cancer [6-17]. In a review by Breslow and Weed, only 6 of 32 studies reported a positive association between alcohol use and prostate cancer [18]; however, they noted that many of the studies had biases that could have attenuated the risk estimates.

Although prostate cancer is known to have a long latency period, lifetime alcohol consumption was not addressed in the studies carried out until the late 1990s, and rarely in the more recent studies [18]. Furthermore, investigators focusing on this topic have considered lifetime alcohol consumption as the average total amount of alcohol consumed over the lifetime, rarely taking into account such characteristics as number of drinks consumed on a typical drinking day or other descriptions of drinking pattern. The distribution of an equivalent volume of alcohol across multiple drinking occasions rather than a single occasion (e.g., one drink per day vs. seven drinks on single day) is likely to have different physiologic effects and impact on cancer risk. Likewise, an examination of average total lifetime alcohol intake does not address the possibility that, although the total lifetime volume may not differ, the duration of intake may, thus effectively resulting in a higher dose over a shorter time period.

Alcohol may act as a carcinogen itself and may also modulate risk from other carcinogen exposures. It has been implicated in risk of cancer at a number of sites [19,20]. In the present case-control study we examined the association between lifetime alcohol intake, duration of alcohol use, and drinks per day with risk of prostate cancer in western New York.

## Methods

We conducted a case-control study of prostate cancer and hormones and alcohol intake (the PROMEN STUDY) in Erie and Niagara Counties, NY, USA, between December 1998 and April 2001. The methods for this study have been previously described in detail [21]. Participants provided informed consent; the Institutional Review Board of the University at Buffalo, School of Medicine and Biomedical Science, and each of the participating hospitals approved the procedures for the protection of human subjects recruited for the study.

Cases were men aged 45 to 85 years with incident, primary, histologically confirmed prostate cancer. Men with a previous history of cancer (except non-melanoma skin cancer), or already on hormonal or chemotherapy treatment (current or in the 6 months prior to diagnosis), as well as those affected by chronic or acute liver diseases, were excluded. Cases aged 35–65 years were also required to have a driver's license, because we used driver's license records to identify age matched controls.

During the study period, 504 men were identified with incident prostate cancer. Of these, 336 men did not meet the eligibility criteria; we invited the remaining 163 patients to participate in the PROMEN study. After being contacted, 50 men refused to participate resulting in a participation rate of 70%. Ninety-six patients had complete data for the variables of interest.

Controls aged between 35 and 65 years were selected from a list of individuals holding a New York State driver's license and residing in Erie and Niagara Counties. Those aged 65 and over were selected from the rolls of the Health Care Financial Administration. As with cases, men on hormonal treatment (current or in the 6 months prior the diagnosis), or diagnosed with metabolic or endocrine disease were excluded, as well as participants with a previous story of cancer other than non-melanoma skin cancer. Since it is well known that latent prostatic carcinoma has a high prevalence in men over 50 [22,23], we evaluated prostate specific antigen (PSA) in the blood samples obtained from controls. Controls found to have a PSA value higher than 4 ng/ml were excluded from the control group, in accordance with the criterion established by the American Cancer Society Prostate Cancer Detection Project [24] until the completion of further diagnostic procedures to clarify their true case-control status.

During the study period, 1373 potential controls were contacted. One hundred and seventy nine of these individuals were deceased or were too ill to participate, 293 did not meet the eligibility criteria and we were not able to contact 272 persons. We identified eight prostate cancer cases as a result of PSA determination in subjects who initially were recruited as controls. Three hundred and seventeen of the remaining 513 subjects (60%) were enrolled and interviewed: 304 had complete data for analysis.

Extensive data on demographics, smoking history, alcohol consumption, and other study variables were collected by trained interviewers during in-person computer-assisted interviews [25] and with self-administered questionnaires. Height, weight, waist and hip circumferences were measured by trained technicians using a standardized protocol. Body mass index (BMI) was calculated as weight in kilograms divided by square of the height in meters (kg/m^2^). Waist to hip ratio (WHR) was calculated as waist circumference divided by hip circumference.

### Alcohol intake

Detailed information on alcohol consumption throughout the lifetime was collected using the Cognitive Lifetime Drinking History [26,27]. Prior to the interview, participants completed a lifetime events calendar on which they recorded the date and their age when significant events in their life occurred. The calendar was used during the interview to help them remember what they were doing during specified periods of their lives and whether drinking alcohol was involved. Participants reported the age when they started drinking alcohol regularly (at least once a month for six months) and when their drinking changed over the years. When changes were reported, participants were asked whether they continued regular drinking; if not, they were asked if they ever resumed regular drinking. Using this information, we defined intervals during each participant's life when drinking patterns were relatively homogeneous and computed the total number of drinking years and the total number of abstinent years. Lists of alcoholic beverages, beer, wine, wine coolers, and liquor, and models of glasses and bottles were used to help respondents recall what beverages they drank over their lifetimes; their usual drink size of each beverage; and whether drink size changed over their lifetimes. This provides information used to: (1) calculate absolute alcohol intakes and (2) tailor the computer-assisted interview to the each respondent's drinking history. Patterns of drinking were ascertained for intervals during which respondents drank weekly or more often by asking how often respondents drank on Fridays, Saturdays, Sundays, and weekdays, and how many drinks they usually had on each. For intervals during which respondents drank less often than weekly, they were asked standard quantity and frequency questions. Quantity and frequency for times when they drank more than usual were assessed for all intervals, as was the frequency of intoxication; the proportion of drinks they had with meals/snack/without eating; and the proportion of drinks from beer, wine, wine coolers, and liquor.

Drinks per interval was estimated by multiplying quantity by frequency for days of the week and more than usual and adding. Drinks per interval was translated into ounces of ethanol per interval based on the proportion of drinks represented by specific beverages, respondents' beverage-specific drink size in ounces, and factors representing the average percent per ounce of absolute alcohol for a given beverage to estimates of drinks per interval. Factors used were 0.048, 0.12, 0.04 and 0.40, for beer, wine, wine cooler and hard liquor, respectively. These estimates were summed across drinking intervals to yield lifetime totals.

We considered several variables in these analyses: total number of years alcohol was consumed, number of drinks per day during the drinking years (total number of drinks/total number of days in drinking years), number of drinks per drinking day (total number of drinks/total number of days on which alcohol was consumed in drinking years), total lifetime ounces of ethanol and beverage-specific total lifetime ounces of ethanol. Because few participants consumed wine coolers, wine and wine coolers were combined. A drink was defined as 12 ounces of beer, 5 ounces of wine, and 1.5 ounces of liquor.

### Statistical analysis

Statistical analyses were conducted using SPSS for Windows version 11.0. Differences between cases and controls in demographic characteristics and alcohol consumption were assessed using t-tests for continuous variables and χ^2 ^for categorical variables. Lifetime abstainers, defined as those subjects who had less than 12 drinks in any one year over their lifetimes, were excluded from our analyses. The biological and social differences between lifetime abstainers and both former and current drinkers [28,29] and the very low number of these subjects in our sample (5 cases and 11 controls) represent the reasons for their exclusion from our analyses. Our final sample size for analysis included 88 cases and 272 controls.

In analyses of risk associated with lifetime alcohol intake, tertiles of total and beverage specific ounces and total drinking years were computed based on the distribution in the controls. For the beverage specific analyses, non-drinkers were those respondents not consuming that particular alcoholic beverage. For risk associated with drinks per day and drinks per drinking day, we categorized consumption as two drinks or less per day and greater than two drinks per day. Odds ratios (OR) and 95% confidence intervals (CI) for risk of prostate cancer associated with alcohol consumption were computed using unconditional logistic regression adjusting for age, cigarette smoking status, education, body mass index (BMI), and waist to hip ratio (WHRATIO). The beverage specific analyses were further mutually adjusted for the other beverages.

## Results

Characteristics of the participants in the PROMEN study are shown in Table 1. Compared to cases, controls were slightly more educated (13.0 vs. 12.3 years) and more likely to be Caucasian (93.0% vs. 67%). No statistically significant differences between cases and controls were observed for age, body mass index, waist to hip ratio, smoking or drinking status.

**Table 1:** Characteristics of prostate cancer cases and controls, PROMEN Study

	Cases (n = 88)	Controls (n = 272)
	Mean (SD^a^)	
Age, years	69.3 (8.4)	70.0 (6.3)
Education, years	12.3 (2.7)^b^	13.0 (2.8)
Body mass index, kg/m^2^	29.2 (5.2)	28.6 (4.6)
Waist to hip ratio		
	Percent	
Race		
White	67.0^c^	93.4
Non white	33.0	6.6
Smoking status^d^		
Never	23.8	28.3
Former	61.4	61.8
Current	14.8	9.9
Drinking status^e^		
Non-current drinkers	36.4	23.5
Current drinkers	63.6	76.5

^a^standard deviation; ^b ^p < 0.05, t-tests for differences in means between cases and controls; ^c ^p < 0.001, χ^2 ^for differences in categorical variables between cases and controls; ^d^smoking status at the time of diagnosis in cases or interview in controls; ^e^drinking status in the 12–24 months prior to diagnosis or interview, non-current drinkers stopped drinking at least 12–24 months prior to interview

Means and standard deviations for aspects of lifetime alcohol consumption for the sample overall and by current drinking status are shown in Table 2. Among drinkers overall and current drinkers, cases drank for fewer years than did controls (38.2 vs. 43.7 years, p < 0.05 and 41.3 vs. 46.8 years, p < 0.05, overall and current drinkers, respectively) and, consequently, had greater numbers of years abstaining. Few differences in lifetime total and beverage-specific ounces consumed, drinks per day, or drinks per drinking day were observed between cases and controls for drinkers overall or current drinkers. However, although not statistically significant, we observed several differences in alcohol consumption between cases and controls who were former drinkers. Among former drinkers, cases consumed more total ethanol, beer and liquor, more drinks per day and more drinks per drinking day, but consumed less ethanol from wine and wine coolers compared to controls.

**Table 2:** Selected lifetime alcohol consumption characteristics among prostate cancer cases and controls, PROMEN study

	All drinkers (n = 360)		Former drinkers (n = 96)		Current drinkers (n = 264)	
	Cases (n = 88)	Controls (n = 272)	Cases (n = 32)	Controls (n = 64)	Cases (n = 56)	Controls (n = 208)

	Mean (SD)		Mean (SD)		Mean (SD)	

Total drinking years	38.2 ^a ^(16.5)	43.7 (14.9)	32.9 (18.5)	33.8 (17.2)	41.3 ^a ^(14.5)	46.8 (12.7)
Total abstaining years	11.4 ^a ^(15.0)	6.6 (12.5)	19.8 (16.4)	18.2 (15.3)	6.6^a ^(11.9)	3.0 (8.8)
Drinks per day	2.6 (7.3)	1.6 (3.4)	4.7 (11.6)	2.5 (5.8)	1.3 (1.7)	1.3 (2.2)
Drinks per drinking day	4.5 (7.3)	3.6 (4.3)	6.8 (11.3)	5.0 (6.3)	3.2 (2.5)	3.2 (3.4)
Total lifetime ethanol, ounces	12904.7 (18681.0)	11735.3 (12904.7)	19051.0 (26382.6)	13498.8 (21019.7)	9392.6 (11187.8)	11192.7 (16880.9)
Total lifetime ethanol from beer, ounces	6282.5 (11321.0)	6024.3 (9250.0)	7771.0 (15173.8)	5992.6 (12284.7)	5431.9 (8422.3)	6034.1 (8129.3)
Total lifetime ethanol from liquor, ounces	5654.2 (14571.6)	4067.2 (12815.8)	10307.0 (22051.6)	5233.7 (11480.5)	2995.5 (6480.2)	3708.3 (13204.5)
Total lifetime ethanol from wine/wine coolers, ounces	953.1 (2715.6)	1634.6 (4168.8)	958.9 (3588.4)	2271.0 (6154.0)	949.8 (2099.5)	1438.8 (3326.0)

^a ^p < 0.05, t-tests for differences in means between cases and controls

Odds ratios and 95% confidence intervals for the risk of prostate cancer associated with lifetime alcohol consumption are shown in Table 3. We observed no associations with risk with lifetime ounces of total ethanol, beer, wine, or liquor. Risk associated with total drinking years, years of abstaining (ever/never), drinking status, drinks per day, and drinks per drinking day are shown in Table 4. Compared to the highest tertile of total drinking years, men in the lowest tertile had a marginally significant increased risk (OR 2.16, 95% CI 0.98–4.78, p for trend <0.05) and, similarly, men reporting ever abstaining compared to those who never abstained had increased prostate cancer risk (OR 1.79, 95% CI 1.05–3.03). No associations with risk were observed for former vs. current drinkers, drinks per day, or drinks per drinking day.

**Table 3:** Odds ratios (OR)^a ^and 95% confidence intervals (CI) for risk of prostate cancer associated with lifetime alcohol consumption

	Cases (n = 88)	Controls (n = 272)	Odds Ratios (95% CI)
Total lifetime ethanol, ounces			
≤2647.62	29	90	1.00
2647.62 – 11048.28	34	90	1.20 (0.65–2.23)
>11048.28	25	92	0.83 (0.43–1.60)
Total lifetime ethanol from beer, ounces^b^			
≤1941.78	42	120	1.00
1941.78 – 6237.30	25	75	1.16 (0.62–2.16)
>6237.30	21	77	0.89 (0.46–1.72)
Total lifetime ethanol from liquor, ounces^b^			
≤932.23	51	152	1.00
932.23 – 3976.79	15	59	0.71 (0.35–1.44)
>3976.79	22	61	0.91 (0.47–1.76)
Total lifetime ethanol from wine and wine cooler, ounces ^b^			
≤511.66	67	177	1.00
511.66 – 2283.00	10	47	0.76 (0.35–1.65)
>2283.00	11	48	0.60 (0.27–1.30)

^a ^Adjusted for race, age (years), smoke, education (years), BMI, WHRATIO; ^b^further mutually adjusted for other beverages

**Table 4:** Odds ratios (OR)^a ^and 95% confidence intervals (CI) for risk of prostate cancer associated with lifetime alcohol consumption: duration, drinking status, drinks per day, and drinks per drinking day.

	Cases (n = 88)	Controls (n = 272)	Odds Ratios (95% CI)
Total drinking years			
>53	14	80	1.00
42 – 53	27	94	1.44 (0.66–3.14)
≤42	47	92	2.16^b ^(0.98–4.78)
Ever abstained from drinking			
never abstained	39	173	1.00
ever abstained	49	99	1.79^b ^(1.05–3.03)
Drinking status ^c^			
current drinkers	56	208	1.00
former drinkers	32	64	1.40 (0.77–2.53)
Drinks per day			
≤2	62	218	1.00
>2	26	54	1.38 (0.76–2.51)
Drinks per drinking day			
≤2	24	106	1.00
>2	64	166	1.57 (0.88–2.79)

^a ^Adjusted for race age, smoke, education (years), BMI, WHRATIO; ^b^p for trend <0.05; ^c^drinking status in the 12–24 months prior to diagnosis or interview. Former drinkers stopped drinking at least 12–24 months prior to interview.

## Discussion

The assessment of lifetime alcohol consumption in cancer etiology has been predominantly expressed through the calculation of either total lifetime volume or an average volume per specified time period across the lifetime. Few investigations have evaluated lifetime drinking patterns in relation to prostate cancer risk. While methodological difficulties challenge the evaluation of drinking patterns, our results suggest that failure to take into account aspects of drinking pattern such as the relative duration and dose of consumption may reduce our ability to clearly elucidate the role alcohol may be playing in cancer development. Although we observed no associations with risk for total lifetime alcohol intake or when alcohol was expressed as average drinks per day or even average drinks per drinking day, our results suggest that the impact may differ when the same volume of alcohol consumption takes place in fewer drinking years over a lifetime.

Furthermore, it is notable that alcohol consumption was much higher among the cases compared with controls who were former drinkers. As alcohol consumption has been positively related to many causes of morbidity, a proportion of these men may have stopped drinking in response to poor health. Whether pre-existing morbid conditions or heavier drinking is related to subsequent development of prostate cancer remains to be clarified.

Our study has several strengths and limitations. A limitation of our study is the small sample size, especially for cases. However, because one of the original aims of the study was an examination of hormones and prostate cancer, both cases and controls were carefully identified. To eliminate the effect on hormone levels by treatment, cases were enrolled in the study prior to starting chemotherapy or hormone therapy; thus increasing the difficulty of case ascertainment. On the other hand, the exclusion of controls with high circulating PSA levels helped to reduce misclassification and to ensure that the control group was free from prostate cancer. The data used in the present analysis were collected as a part of an in-person interview, and the questionnaire about lifetime alcohol consumption was very detailed allowing us to compute both the dose and frequency aspects of alcohol consumption.

Given the difficulties involved in measuring alcohol consumption, studies utilizing data collected before diagnosis would appear more likely to lead to valid inferences. Recently, Dennis in his meta-analysis [30] pointed out that in many of the published cohort studies alcohol consumption was assessed only at a baseline, often many years before the diagnosis, with no subsequent assessment to quantify changes in drinking pattern. While retrospective assessment of lifelong alcohol consumption at diagnosis may appear to be more likely to lead to recall bias, such an assessment may also be more likely to capture relevant attributes of exposure, such as overall duration of alcohol use and timing of potentially important changes in use, such as quitting. These differences are not always taken into account [30].

The plausibility of alcohol as a risk factor for prostate cancer relates to evidence that alcohol may act as a carcinogen or may modulate risk from other known carcinogens through generation of free radicals, affecting the metabolism of detoxification enzymes, impairment of immune system and depression of DNA repair enzymes [31]. It remains unclear to what extent alcohol could affect the early phases of cancer development. Some studies suggest that the critical period of exposure may be as early as adolescence as the development of prostate gland begins prenatally, continuing until the end of puberty [31]. If alcohol contributes to cancer promotion, duration and relative intensity of exposure during a specified period of time, instead of the total amount of the agent itself over the entire life time course may be important.

## Conclusions

Further studies focusing on lifetime exposure and more specifically on patterns of consumption may help in prevention of a disease with considerable public health impact.

## List of Abbreviations

BMI body mass index

PSA prostate-specific antigen

WHRATIO waist-to-hip ratio

## Competing interests

The authors declare that they have no competing interests.

## Authors' contributions

MB performed statistical analyses, interpreted the results and drafted the manuscript

SEM performed statistical analyses, interpreted the results and revised the manuscript

HJS interpreted the results and revised the manuscript

SS interpreted the results and revised the manuscript

BJF performed data analysis, interpreted the results and revised the manuscript

SDP interpreted the results and revised the manuscript

GC interpreted the results and revised the manuscript

JLF interpreted the results and revised the manuscript

MT revised the manuscript

MR defined the exposure variables and revised the manuscript

TN defined the exposure variables and revised the manuscript

PM designed and implemented the study, interpreted the study results, revised the manuscript
